# A Functional Magnetic Resonance Imaging Approach for Language Laterality Assessment in Young Children

**DOI:** 10.3389/fped.2020.587593

**Published:** 2020-11-17

**Authors:** Lisette Charbonnier, Mathijs A. H. Raemaekers, Philippe A. Cornelisse, Maxime Verwoert, Kees P. J. Braun, Nick F. Ramsey, Mariska J. Vansteensel

**Affiliations:** ^1^Department of Neurology and Neurosurgery, UMC Utrecht Brain Center, University Medical Center Utrecht, Utrecht, Netherlands; ^2^Department of Child Neurology, UMC Utrecht Brain Center, University Medical Center Utrecht, Utrecht, Netherlands

**Keywords:** fMRI, children, language, mapping, lateralization

## Abstract

Functional magnetic resonance imaging (fMRI) is a usable technique to determine hemispheric dominance of language function, but high-quality fMRI images are difficult to acquire in young children. Here we aimed to develop and validate an fMRI approach to reliably determine hemispheric language dominance in young children. We designed two new tasks (story, SR; Letter picture matching, LPM) that aimed to match the interests and the levels of cognitive development of young children. We studied 32 healthy children (6–10 years old, median age 8.7 years) and seven children with epilepsy (7–11 years old, median age 8.6 years) and compared the lateralization index of the new tasks with those of a well-validated task (verb generation, VG) and with clinical measures of hemispheric language dominance. A conclusive assessment of hemispheric dominance (lateralization index ≤-0.2 or ≥0.2) was obtained for 94% of the healthy participants who performed both new tasks. At least one new task provided conclusive language laterality assessment in six out of seven participants with epilepsy. The new tasks may contribute to assessing language laterality in young and preliterate children and may benefit children who are scheduled for surgical treatment of disorders such as epilepsy.

## Introduction

Adequate functional brain mapping is especially relevant for children and adults for whom resective neurosurgery is considered, such as people with epilepsy (1). Resective brain surgery aims to take away the diseased tissue without damaging the eloquent cortex since damage to these areas would result in significant motor and/or language impairment of the patient (2). Importantly, in the treatment of pediatric epilepsy, there is an increasing tendency to perform such resective surgery early since timely intervention benefits long-term seizure and cognitive outcome (3, 4). One of the most frequently addressed questions in preoperative function mapping is that of language lateralization (5), under the rationale that if surgery needs to be performed on the language-dominant hemisphere close to the language areas, additional measures are needed to more precisely localize these areas [such as electrical stimulation mapping (6)], whereas surgery in the non-dominant hemisphere does not require this extra information. An answer to this question is relevant for pediatric epilepsy surgery for several reasons. First, there is evidence that language lateralization in the brain already appears early in life (7). Second, there is a relatively large incidence of atypical language lateralization among patients with epilepsy, including children (8, 9), which is thought to be related to the chronic nature of the condition (10). The intracarotid amobarbital test has long been considered as the gold standard for assessment of language lateralization, but since that procedure is highly invasive and stressful (11), other methods are investigated, including magnetoencephalography [e.g., (12)], transcranial magnetic stimulation [e.g., (13, 14)], and functional transcranial doppler [e.g., (15)].

One of the most extensively examined techniques to determine lateralization and more detailed spatial localization of brain functions, including language, is functional magnetic resonance imaging (fMRI). Its non-invasiveness, safety, high spatial resolution, and wide availability of the required equipment have made fMRI a valuable technique to study brain function in both research and clinical settings [e.g., (16)]. For high-quality fMRI images, optimal patient or participant compliance is crucial, which for adults is generally accomplished by simple instructions. However, the increasing number of fMRI studies in children face important challenges, causing lower success rates of fMRI scans [e.g., incomplete and/or low-quality datasets; (17–19)]. These challenges include lack of cooperation (e.g., fear to enter the scanner or requests to prematurely exit the scanner), suboptimal understanding of the instructions or inability to perform the tasks, and excessive head motion (18, 20, 21). Especially in younger children, more so in boys than in girls and more so in patients than in healthy participants, these issues play a role and may lead to large percentages of failed scans (17–19). Sedation can be a solution to address anxiety and head motion but has clear disadvantages in terms of patient burden, risk, and expense and is not compatible with the need for alertness and performance of a task in most fMRI sessions. Resting-state fMRI may solve the task compliance issue and has shown promising results for language laterality assessment in several studies on both adults and children (>10 years) with epilepsy (22–24). Others have reported, however, that data acquired during resting state was associated with more head motion than data acquired during task performance (25) and that concordance between the lateralization index as determined with task-fMRI and with resting-state fMRI was moderate (26). Evidence for the value of resting-state fMRI in *young* children with epilepsy is still extremely scarce [e.g., (27)], and the value of this technique for this young group of patients still remains to be determined before it can be applied in clinical settings.

As a first step toward improved functional mapping in clinical pediatric populations, we here aimed to develop and validate an fMRI approach suitable for language mapping in young children, including those who are not yet able to adequately read words. To maximize participant cooperation and minimize head motion during scanning, we designed two new child-friendly language tasks, which were not only suitable for young age in terms of difficulty but which also aimed to attract attention and keep children entertained by making use of the concepts of story listening and response feedback. The activation patterns of the new tasks were compared with those of a well-validated task for language mapping in a group of healthy children. In addition, we applied the tasks in a consecutive group of children with epilepsy and compared language lateralization as assessed with the new tasks with the results obtained using other clinical measures.

## Materials and Methods

### Ethical Statement

The study was approved by the Medical Ethical Committee of the UMC Utrecht and was carried out in accordance with the Declaration of Helsinki (2013). The parents of all participants gave written informed consent for their children to participate.

### Healthy Participants

The healthy participants (*n* = 32, 15 male, 17 female, 27 right-handed, five left-handed, 6–10 years old) were native Dutch speakers, but one of them was bilingual. Although the new tasks were designed to be suitable for children who are unable to read, we aimed to compare the activation patterns of the new tasks with those of a well-validated task for language lateralization assessment that requires reading ability and therefore did not screen or pre-select children on their level of cognitive development and reading or language skills. One child was reported to be dyslexic. Participant screening involved a question about medication and a question about health (“Does your child have health problems or abnormalities that may be relevant to the study?”). None of the children had medication or health issues that were considered relevant for the study. Exclusion criteria were irremovable metal objects on or inside the body and anxiety in the scanner, as evaluated by the parents during screening or based on the outcome of the mock-scan procedure.

### Participant Assessment and Preparation

Participant preparation was performed in a dedicated room that was equipped with a full-scale mock-scanner. One or both parents were present during the preparation procedure. After informed consent, the participants filled out the Edinburgh Handedness Inventory (28) and an fMRI safety screening form. Subsequently, the fMRI tasks were explained and practiced using a laptop computer. Finally, children were habituated to the scanner environment using the mock-scanner. After explaining the different parts and the necessity of lying still, children were invited to lie down on the mock-scanner bed and were equipped with earplugs, earphones, and head-coil. The different scanner sounds were played with increasing volume. Then, children were moved step by step into the bore of the mock-scanner. Once inside the bore, the different scanner sounds were repeated with increasing volume.

Before and after the mock-scanner practice, the participants, the parents, and the researcher filled out two Visual Analog Scales (VAS) to evaluate (1) how much anxiety they/the participant felt about participating in the fMRI experiment and (2) how enjoyable they/the participant considered participation. Scales ran from 1 (not anxious, very enjoyable) to 10 (very anxious, not at all enjoyable). Care was taken that the participants, the parents, and the researcher were not aware of each other's ratings. In addition, to avoid any bias, we did not explicitly discuss that we were interested in the effect of the mock-scanner practice on the anxiety and enjoyment scores. After the actual fMRI scan, the participants repeated the VAS.

Notably, the post mock-scan researcher ratings of one participant and the post-fMRI scan ratings of another participant were inadvertently not filled out. Four participants had had a recent fMRI scan for another project. Given their experience with the fMRI setting, a mock-scan session was not performed with these children, but tasks were practiced.

#### Analysis of VAS Results

VAS scores obtained before and after the mock-scanner practice were compared with repeated-measures ANOVA. The post-fMRI VAS scores of the participants were compared with their values before and after the mock-scan session using repeated-measures ANOVA.

### Tasks

We developed two child-friendly tasks for language mapping (story task and letter picture matching task), both targeted at children of young age and designed to automatically attract their attention and keep them entertained. The results of these tasks were compared with those of the classic verb generation language task (29). The tasks were performed in random order.

#### Story Task

The story (SR) task is a receptive language task with a visual component and reversed speech (indicated by the “R” in the task abbreviation) as a control condition. Reversed speech has similar acoustic characteristics as speech and therefore has been proposed as a suitable control condition to separate the language-specific features of speech from basic auditory aspects (30, 31). The SR task is based on the principle of reading a picture book to a child, where illustrations support the narrative and attract the child's attention. The SR task design was as follows: Children listened to a female voice of a speech and language therapist reading an adapted (i.e., shortened) version of a children's book story (task duration 9.3 min). During each active condition (speech, *n* = 14 blocks, 8.7–38.6 s in duration, total 315 s), the children were visually presented with a colorful illustration that supported the narrative. During each rest condition (reversed speech, *n* = 14 blocks, 16.6–19.1 s in duration, total 245 s), the children heard reversed speech and watched the illustration slowly turning (like the page of a book) to the next illustration that supported the narrative of the subsequent excerpt (page turning lasted as long as each reversed speech condition) of the story. In other words, during the rest condition, the children saw the illustration of the previous excerpt slowly being replaced for the illustration of the next. During task practice, the children were informed about the fact that the sound during the control condition would be unrecognizable, and they were instructed to just watch the page turning. Sound was delivered through a dedicated MRI-compatible audio system with in-ear plugs (MR Confon, Magdeburg, Germany). The children were instructed to press the alarm button if they wanted to have the audio volume adjusted.

#### Letter Picture Matching Task

The letter picture matching (LPM) task combines the concepts of picture naming and letter naming (32), was modified from the letter task reported by Wilke et al. (33), and combines both letter recognition and naming concepts. The participants were visually presented with a letter for 2 s. Next, three pictures (34) were shown sequentially (2 s each). The instruction was to evaluate, for each picture, whether the object started with the letter just presented and to respond by squeezing a balloon with their dominant hand if it did. A squeeze was rewarded with a colored line around the picture. For each set of three pictures, only one picture was correct (i.e., the target for a squeeze), but this was not explicitly mentioned during instruction. Each active block contained two trials, both consisting of a letter and three pictures that had to be evaluated against that letter. Trials in rest blocks started with the hash sign, followed by three scrambled pictures, one of which was marked by a clear horizontal, vertical, or diagonal line (the target for a squeeze). Each rest block contained two trials. The task consisted of 12 active blocks and 12 rest blocks. Total task duration was about 7 min. Response accuracy of this task was defined as the percentage of targets that was responded to with a balloon squeeze (true positive responses).

#### Verb Generation Task

The verb generation (VG) task is known to reliably lateralize language representation in both adults and compliant children aged 7 and older [e.g., (35, 36)]. It is the most frequently used task for the assessment of language lateralization in clinical settings (37) and shows high concordance with invasive approaches, including the intracarotid amobarbital (wada) test and electrocortical stimulation mapping (36, 38, 39). However, since it was originally designed for adults, it has limited value in terms of entertainment or attracting the attention of children. In addition, we have noted that young children with epilepsy are sometimes unable to read and produce words at sufficient speed. The task design was as follows: during active blocks, nouns were presented on the screen (as written words, 1 s per noun, nine nouns per block), followed by a fixation cross (2 s). The participants were instructed to generate a verb that was related to the presented noun and covertly pronounce this verb. During the rest condition, the participants were shown symbols instead of nouns (e.g., “/”) and were instructed to relax and think of nothing in particular. Pauses between active and rest blocks were slightly longer than 2 s. Total task duration was 4.9 min (five active and five rest blocks). For three children, a slower version of the VG task was used because, during the preparation, it became clear that they were unable to keep up with the pace of the normal version of the VG task. In the slow VG task, nouns (six per block) were presented for 4 s. The number of active and rest blocks was the same as in the regular task.

### Functional MRI Data Acquisition

Functional MRI data were acquired on a 3T scanner (Philips Achieva, Best, The Netherlands). We used PRESTO, a technique that optimizes localization accuracy by minimizing the confounding effects of large blood vessels (40–42). The fMRI scan parameters were repetition time = 22.5 ms, echo time = 31.22 ms, 0.608 s per volume, flip angle 10°, voxel size 4 mm isotropic, 40 slices, field of view 224 × 256 × 160 mm, prescribed sagittal, ear to ear. For each participant, a T1-weighted structural MRI scan was acquired as well (1 mm isotropic). During the acquisition of the T1-weighted structural MRI scan, the children watched a video of their choice.

### Functional MRI Data Analysis

The fMRI data were analyzed offline with SPM12 (http://www.fil.ion.ucl.ac.uk/). First, functional images were realigned to the first scan and then co-registered to the individual T1-weighted scans using a reference scan, normalized to MNI space, and smoothed with a Gaussian kernel (8 mm full width at half-maximum). Statistical analysis involved performing a general linear model (GLM) on the data and the generation of contrast maps. Motion correction was accomplished by adding two confound factors to the GLM, namely, (1) the six realignment parameters, as produced by SPM12 (http://www.fil.ion.ucl.ac.uk/spm/software/spm12/) in the realignment preprocessing step, which include the *x, y*, and *z* rotations and translations necessary to align each functional volume with the first acquired volume, and (2) a motion filter. The motion filter consisted of a set of finite impulse response functions that were added to the design matrix and effectively removed the contribution of images during which excessive head motion had occurred. Notably, the motion filter approach effectively removes the contribution of images with excessive head motion. Image removal may result in loss of power due to a decrease in the number of images and due to a change in the distribution of images between active and rest blocks (e.g., when one of the conditions contains more movement than the other). To establish the proportion of statistical power (PSP) remaining after the addition of the motion filter, we used the following formula:

$$\begin{array}{l} \text{PSP}=\frac{R_{m}^{2}\times \sqrt{df_{m}}}{R^{2}\times \sqrt{df}} \end{array}$$

where $R_{\text{m}}^{2}$ and *R*^2^ are the multiple correlation coefficients between the task-factor and the remaining factors in the design matrix for the design with and the design without the motion filter, respectively; *df*_m_ and *df* are the degrees of freedom of the design with and without motion filter, respectively. Datasets were excluded if the PSP was lower than 0.4.

The single-subject contrast maps were entered into a second-level analysis (one-sample *t*-test) to obtain the group maps (right-handed participants only, to avoid the inclusion of right-lateralized language patterns in the mean activation pattern). A cluster level threshold of *p* < 0.05 corrected for multiple comparisons across the mask volume was derived using Monte Carlo simulations (10,000 iterations) of random noise distribution across the whole brain using the 3dClustSim tool in AFNI version 16.2.07 (43, 44). This approach combines an individual voxel probability threshold with a minimum cluster size to estimate the probability of a false positive. We used the 3DFWHMx tool in AFNI to estimate the noise smoothness values of the data using the auto-correlation function option. The resulting two-sided threshold was *p* < 0.001, with a cluster extent *k* ≥ 30 (SR), *k* ≥ 33 (LPM), and *k* ≥ 40 (VG), respectively, for the whole brain.

### Language ROI

Since the three language tasks under study are different regarding aspects other than language, they can be expected to co-activate brain areas other than language regions to a different degree. We therefore only considered the inferior frontal and temporal language areas in the comparison of the lateralization index between tasks as discussed below. To this purpose, a language region of interest (ROI) was produced using the automated anatomical labeling atlas (45), containing the opercular, triangular, and orbital parts of the inferior frontal gyrus (Frontal_Inf_Oper; Frontal_Inf_Tri; Frontal_Inf_Orb), rolandic operculum (Rolandic_Oper), insula (Insula), supramarginal gyrus (Supramarginal), angular gyrus (Angular), Heschl's gyrus (Heschl), and the middle and superior temporal gyrus (Temporal_Mid; Temporal_Sup). In addition, because of their different concepts, the three tasks may activate different parts of the language network to a different extent. Therefore, we compared the number of significantly activated voxels between the three tasks [family-wise error (FWE)-corrected, *p* < 0.05; right-handed participants only] in each of the two main language areas of each participant, namely, Broca's area (bilateral opercular and triangular parts of the inferior frontal gyrus) and (extended) Wernicke's area (middle and superior temporal gyrus, angular gyrus, supramarginal gyrus).

### Lateralization Index

For the computation of the lateralization index (LI), we used a threshold-independent method (46). Shortly, for the section Language ROI (described in section Functional MRI Data Analysis) of the left and the right hemispheres, we calculated the product between the height of the bins of the histogram of the voxels' *t*-values (*t*-value range 0–∞, bin size 0.25) and the square of the index of the bins so that the voxels with higher *t*-values were assigned a heavier weight. The resulting areas under the curve for the left and the right hemisphere were then used in the LI computation. We considered the assessment of LI as conclusive when LI values were ≥0.2 or ≤-0.2, with LI values ≥0.2 being conclusively left-lateralized and LI values ≤-0.2 being conclusively right-lateralized. LI values between −0.2 and 0.2 were considered inconclusive. We chose this value as a cutoff since it is frequently used in other fMRI studies on language laterality assessment (11). To compare the LI results between tasks, we only considered participants (both left- and right-handed) who performed the VG task *and* one (or both) of the new tasks. We report the percentages of participants for whom the new tasks provide the same conclusion on hemispheric language dominance as the VG task. LI values were compared across tasks using paired *t*-tests.

### Clinical Validation

As a first step in the clinical validation of the new fMRI tasks, we investigated (1) whether or not children with epilepsy were able to complete the new tasks in the MRI scanner, (2) if the new tasks provided conclusive LI values in children with epilepsy, and (3) if the language laterality assessment of the new tasks in children with epilepsy corresponded with that of the VG task and/or with other clinical measures for language laterality assessment. To this purpose, children with focal epilepsy who were being evaluated for epilepsy surgery (*n* = 7, five male, two female, five right-handed, two left-handed, 7–11 years old) performed an fMRI scan session in which they performed the SR task and, time and capability permitting, the LPM and the normal or the slow version of the VG task. All children were assessed and prepared using similar procedures as the healthy children, although a mock-scan session was not used for four children given their extensive experience with MRI scans. Analysis of the fMRI data was conducted in single-subject space (normalization was not applied) as specified above. Subsequently, when available, information about language dominance obtained with other clinical measures was compared to the language laterality as assessed with the new tasks.

## Results

### Participant Preparation

**Figure 1:**
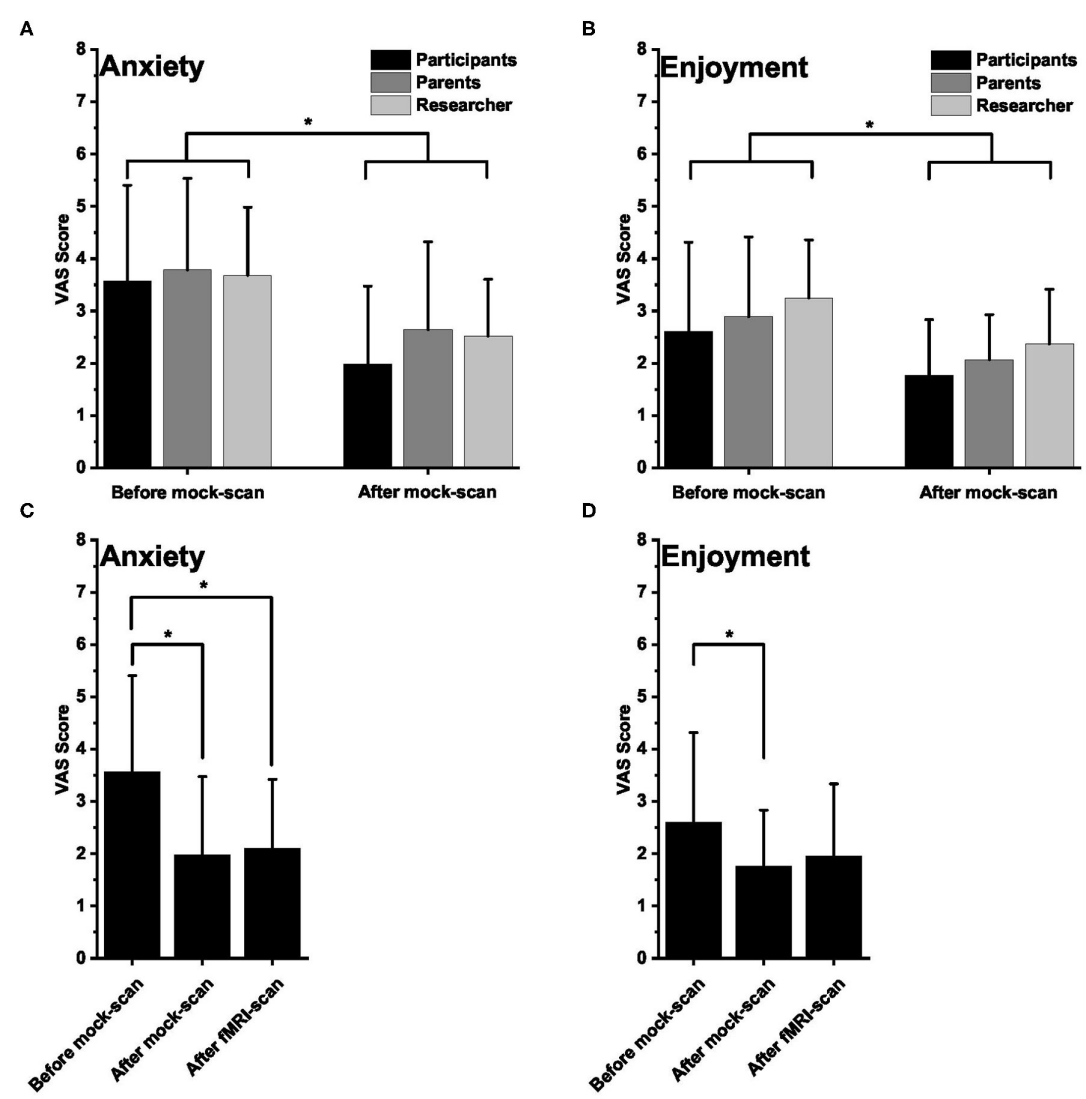
Visual Analog Scale (VAS) scores. **(A**,**B)** Mean (±SD) VAS scores for anxiety **(A)** and enjoyment **(B)** as rated by the healthy participants, their parent, and the researcher before (*n* = 28; 28; 28, respectively) and after (*n* = 28; 28; 27, respectively) the mock scan session. For anxiety, lower scores mean lower anxiety. For enjoyment, lower scores mean more enjoyable. **(C**,**D)** Mean (±SD) VAS scores for anxiety **(C)** and enjoyment **(D)** as rated by the participants before and after the mock scan session and after the functional magnetic resonance imaging session. In all panels, an asterisk indicates a significant difference.

In general, the healthy participants were not very anxious and enjoyed participating in the study, as indicated by the low mean VAS scores reported by the participants, the parents, and the researchers (Figure 1). The VAS scores for anxiety and enjoyment were significantly lower after the practice session than before the practice session, which means that healthy children perceived less anxiety and increased enjoyment after the mock-scanner practice [repeated-measures ANOVA, significant effect of mock-scanner practice for anxiety *F*_(1, 26)_ = 28.182, *p* < 0.001, and enjoyment *F*_(1, 26)_ = 17.150, *p* < 0.001; Figure 1]. For enjoyment, there was an additional effect of group [*F*_(2, 25)_ = 7.611, *p* < 0.01]. *Post-hoc* pairwise comparisons revealed that the enjoyment VAS scores reported by the researchers were significantly higher (i.e., less enjoyment) than those reported by the participants [*p* < 0.05, FDR-corrected for multiple comparisons; (47)].

The participants (not parents and researchers) also filled out the VAS scores after the fMRI scan, which were compared with the VAS ratings before and after the mock-scan session. There was a significant effect of time for both anxiety [repeated-measures ANOVA, *F*_(2, 25)_ = 9.879, *p* < 0.01] and enjoyment [repeated-measures ANOVA, *F*_(2, 25)_ = 5.312, *p* < 0.05]. *Post-hoc* pairwise comparisons revealed that, for anxiety, the post-fMRI VAS scores were not different from those after the mock-scan session and remained significantly different (*p* < 0.05, FDR-corrected for multiple comparisons) from the pre-mock-scan values. For enjoyment, *post-hoc* pairwise comparisons showed that the post-fMRI VAS scores were not significantly different from either the post-mock-scan values or from the pre-mock-scan scores. There was a significant difference between the pre- and the post-mock-scan values however (*p* < 0.05, FDR-corrected for multiple comparisons; Figure 1).

### Tasks and Performance

For 30 of the 32 included healthy participants (94%), aged 8.4 ± 1.4 (mean ± SD) years old (Supplementary Table 1), at least an anatomical scan and one functional scan was acquired. One participant was uncomfortable in the scanner (mainly related to noise) and requested to leave the scanner shortly after scanning started, and for one participant, no data were acquired due to technical issues with the MRI scanner. The total number of data sets per task, as well as the number and the identity of the tasks acquired per healthy participant, varied (see Supplementary Table 1 for details).

For the LPM task, performance by healthy children was generally adequate, with a mean (± SD) accuracy of 86 ± 12% (*n* = 23) correct target selections. For three children, performance was lower than 70%. Considering the number of times these children squeezed the response balloon and the distribution of the squeezes over the active and the rest conditions, however, we concluded that they were actively involved in the language task and therefore did not exclude their data based on their performance. Inherent to the VG and the SR task designs, quantification of performance was not possible for these tasks.

### Motion Correction

The motion filter removed 13.9 ± 15.3% (SR, *n* = 23; mean ± SD), 16.1 ± 19.1% (LPM, *n* = 23), and 10.3 ± 15.1% (VG, *n* = 24) of the scans due to excessive head motion. There was no significant difference between tasks in the percentage of removed scans [one-way ANOVA, *F*_(2, 65)_ = 0.761, *p* = 0.471]. For three datasets (one for each task), the motion filter did not identify any scans with excessive head motion since the participants hardly moved in the scanner (Supplementary Table 2). For two datasets of the LPM task and one dataset of the VG task, the PSP of the remaining scans was lower than 0.4, and these datasets were therefore removed from further analysis (Supplementary Table 2). The mean (± SD) PSP values of the remaining datasets (right- and left-handed participants) were 0.86 ± 0.16 (SR, *n* = 23), 0.88 ± 0.12 (LPM, *n* = 21), and 0.92 ± 0.11 (VG, *n* = 23). There was no significant difference between tasks for these PSP values [one-way ANOVA, *F*_(2, 64)_ = 1.193, *p* = 0.310].

### Group Maps

**Figure 2:**
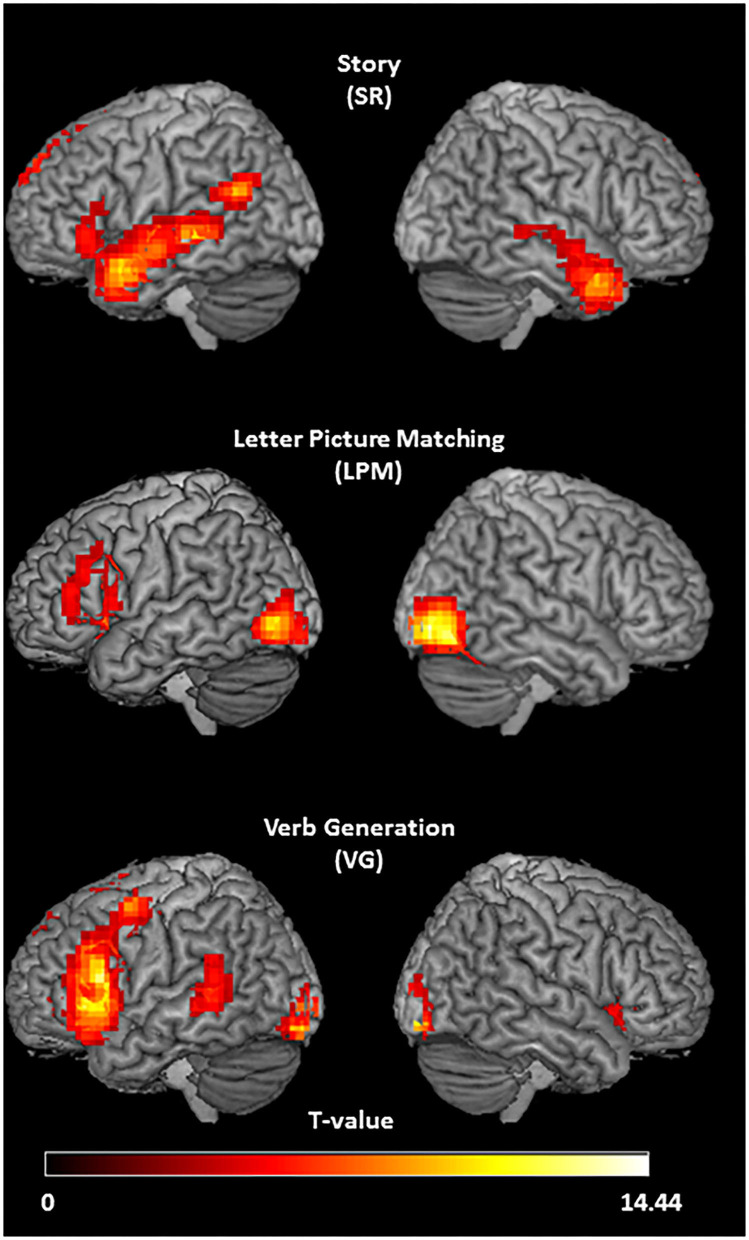
Activation patterns of the three tasks. Group activation t-maps of the three language tasks in right-handed healthy participants, showing stronger activation in response to listening to speech vs. reversed speech (story task; *N* = 20; *T* = 3.58; *p* < 0.001, threshold extent *k* ≥ 30), letter picture matching vs. scrambled picture matching (letter picture matching task; *N* = 17; *T* = 3.69; *p* < 0.001; threshold extent *k* ≥ 33), and verb generation vs. symbol viewing (verb generation task; *N* = 20; *T* = 3.58; *p* < 0.001, threshold extent *k* ≥ 40).

The group activation pattern of the SR task (*n* = 20, right-handed participants only, Figure 2) showed left-lateralized activation in the inferior frontal gyrus. Temporal cortex activation was extended, with bilateral anterior temporal activation and left-lateralized activity in the middle temporal gyrus and posterior superior temporal gyrus/angular gyrus. In the superior frontal gyrus, left lateralized activity was also found, and the posterior cingulate cortex showed activation as well. The LPM task (*n* = 17, all right-handed, Figure 2) activated a large area in the left inferior frontal sulcus, a region in the left medial frontal gyrus, and the bilateral occipital areas. The VG task (*n* = 20, all right-handed, Figure 2) showed highly left-lateralized activation in the typical language areas of the inferior frontal gyrus and the posterior middle temporal gyrus. Activity was also present in the left precentral gyrus, the left superior and medial frontal gyrus, and the bilateral occipital areas.

**Figure 3:**
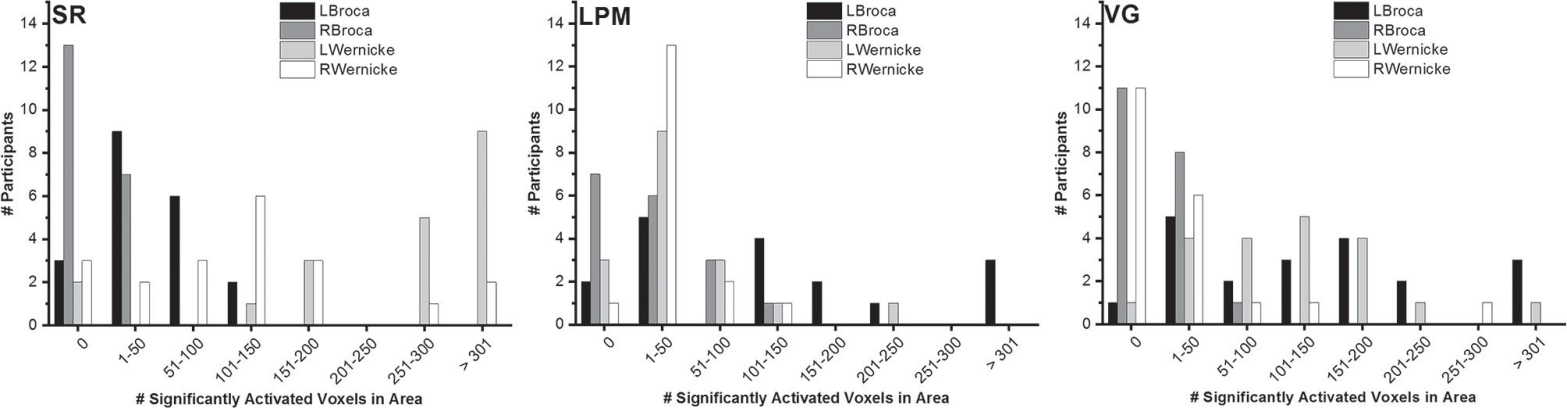
Number of significantly activated voxels. Distribution of the number of significantly activated voxels in Broca's and Wernicke's areas, over right-handed healthy participants (story: *n* = 20; letter picture matching: *n* = 17; verb generation: *n* = 20) and per task.

The number of significantly activated voxels in Broca's and Wernicke's areas showed considerable variability between participants (Figure 3). All tasks did, however, activate both Broca's and Wernicke's areas to a certain extent in most participants. For Broca's area, the total number of right-handed participants with at least one significantly activated (FWE-corrected, *p* < 0.05) voxel in the left hemisphere was 17/20 (SR), 15/17 (LPM), and 19/20 (VG). For Wernicke's area, these numbers were 18/20 (SR), 14/17 (LPM), and 19/20 (VG).

### Lateralization Index

**Figure 4:**
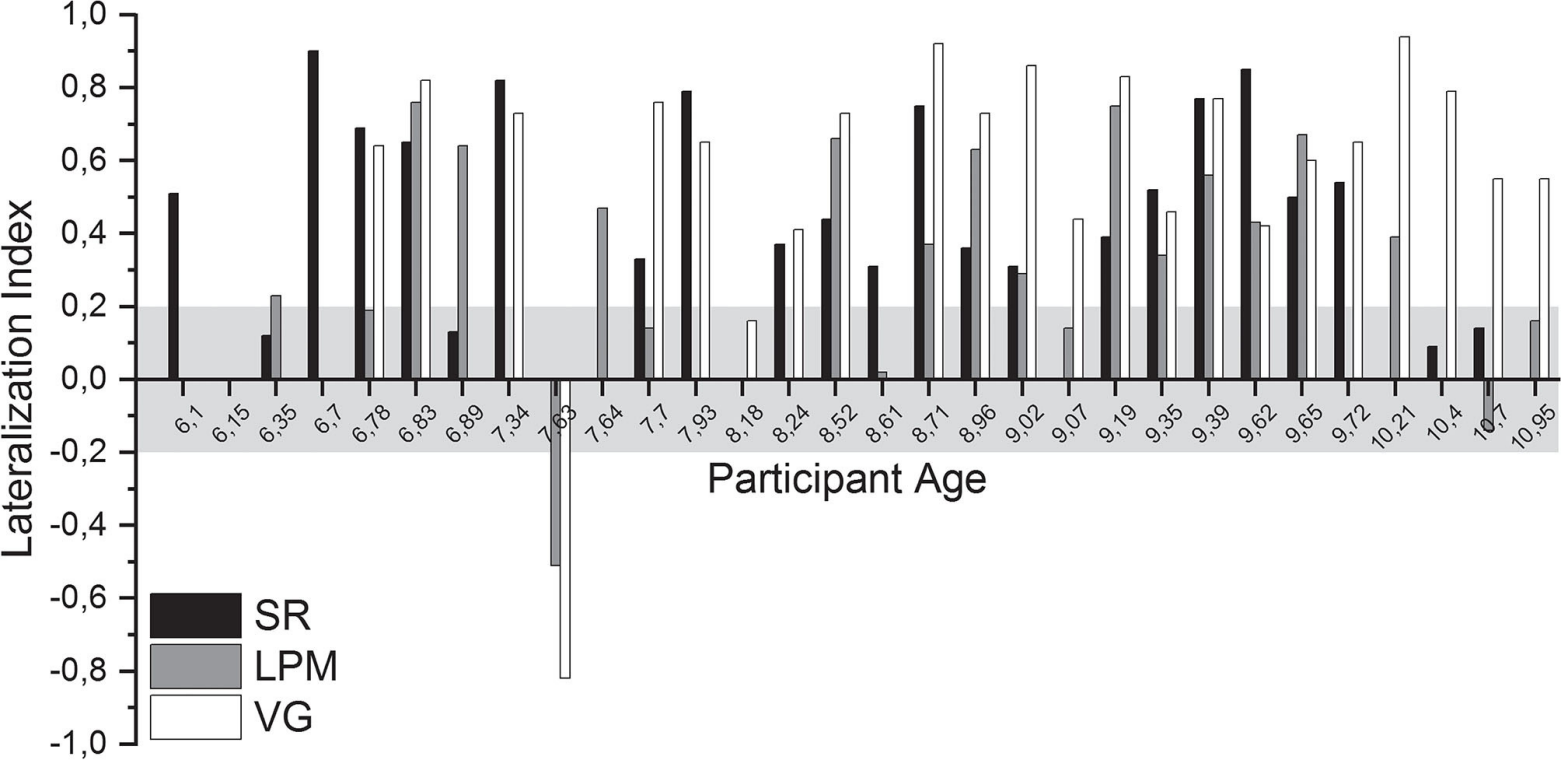
Lateralization indices. Lateralization index (LI) of all language tasks of all healthy participants, ordered by participant age. Gray shading indicates the range of LI values that was considered inconclusive (i.e., between −0.2 and 0.2).

The mean (±SD) LI of right-handed participants was 0.51 ± 0.25 (SR, *n* = 20), 0.37 ± 0.27 (LPM, *n* = 17), and 0.65 ± 0.20 (VG, *n* = 20; see Figure 4 and Supplementary Table 3 for individual values). For left-handed participants, the mean LI values were 0.34 ± 0.21 (SR, *n* = 3), 0.21 ± 0.50 (LPM, *n* = 4), and 0.19 ± 0.87 (VG, *n* = 3).

For 28 of the 30 participants (93%) for whom functional data were acquired, a conclusive lateralization index (i.e., ≤-0.2 or ≥0.2) was obtained for at least one of the performed tasks. Per task, conclusive lateralization indices were obtained for 19/23 (83%, SR), 15/23 (65%, LPM), and 22/24 (92%, VG) of the participants (Supplementary Table 3).

For 15 of the 16 (94%) children who performed *both* the SR and the LPM tasks, a conclusive lateralization index was obtained for at least one of the tasks (one task conclusive: *n* = 5; both tasks conclusive: *n* = 10; Supplementary Table 3).

#### Comparison of LI Results Between Tasks

Only healthy participants who performed the VG task *and* at least one of the new tasks (SR and LPM) were included in the direct comparison of lateralization index, thereby allowing for within-subject comparisons of the results of the new tasks compared to the VG task. Importantly, for none of the participants, the new tasks conclusively lateralized language to the other hemisphere as that concluded from the VG task.

A comparison of the LI results of the SR task to those of the VG task revealed that, for 16 out of 19 (84%) children who performed both tasks, the results were consistent in showing left-lateralized language. For two other participants, the LI based on the SR task was inconclusive, whereas the VG task indicated left-lateralized language. For another participant, the VG data were excluded because of excessive motion, and the SR task lateralization index was inconclusive. For the participants with usable data from both tasks, the mean LI was significantly lower for the SR task than for the VG task, both when right- and left-handed participants were included in the analysis (*n* = 18, 0.52 ± 0.23 and 0.68 ± 0.15, respectively, paired *t*-test, *p* < 0.05) and when only right-handed participants were considered (*n* = 16, 0.53 ± 0.24 and 0.68 ± 0.16, paired *t*-test, *p* < 0.05).

For the LPM task, a comparison with the VG task revealed that, for 12/19 (63%) participants who performed both tasks, LPM and VG indicated similar language lateralization (11 left-lateralized language and one right-lateralized language). For five other participants, the LPM task was inconclusive, whereas the VG task indicated left-lateralized language. For one participant, the VG data were excluded due to head motion, whereas the LPM task showed conclusive lateralization. For one other participant, the LPM data were excluded, and the VG lateralization index was inconclusive. Mean LI was significantly lower for the LPM task than for the VG task for the participants with usable data from both tasks (right- and left-handed: *n* = 17, 0.35 ± 0.33 and 0.60 ± 0.40, respectively, paired *t*-test, *p* < 0.01; only right-handed: *n* = 15, 0.38 ± 026 and 0.69 ± 0.18, paired *t*-test, *p* < 0.01).

### Clinical Validation

The regular version of the VG task was performed by three of seven participants with epilepsy, two of whom requested the task to be suspended prematurely. For the other four of seven participants with epilepsy, the regular version of the VG task was too difficult. Two of them were able to perform the slow version of the VG task. In contrast, the SR task was completed by six of seven participants with epilepsy and partially completed by the 7th participant with epilepsy. The LPM task was performed by five of seven participants with epilepsy. Two participants did not conduct the LPM task due to time constraints. None of the acquired datasets had to be excluded due to excessive head motion (all PSP were >0.4). Performance during the LPM task was 72 ± 23%, with two participants (EP3 and EP4) scoring lower than 70% correct.

**Figure 5:**
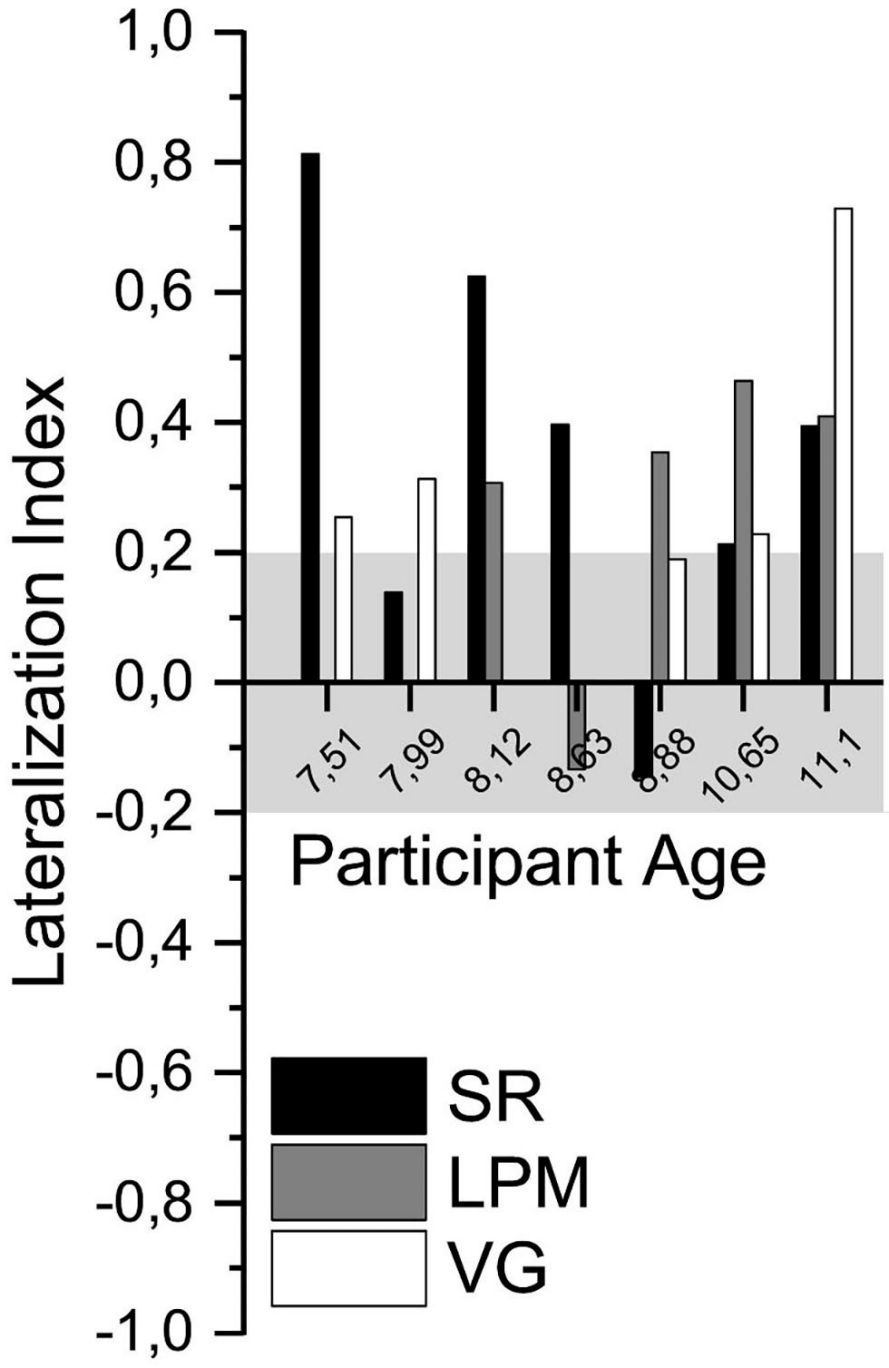
Language lateralization in participants with epilepsy. Lateralization index of all language tasks of the participants with epilepsy, ordered by participant age. Gray shading indicates the range of LI values that was considered inconclusive (i.e., between −0.2 and 0.2).

Per task, language lateralization was conclusive for five of seven (71%, SR), four of five (80%, LPM), and four of five (80%, VG) participants with epilepsy, respectively (Figure 5). The one participant with inconclusive LPM lateralization had very poor performance (EP4, 35% correct).

For the two participants with epilepsy who were unable to perform the VG task (regular nor slow), the SR task resulted in a conclusive LI value, and for one of them the LPM task was also conclusive. Three participants with epilepsy performed all three tasks. In one of these participants, the VG task LI value was not conclusive, but the LPM task conclusively lateralized language to the left hemisphere. For the other two participants, one or both of the new tasks conclusively lateralized language to the left hemisphere, which corresponded with the results of the VG task. The two other participants with epilepsy performed the VG and the SR tasks. In one of them, the VG and the SR LI values were conclusive and in the same direction. The SR LI value of the other participant was inconclusive.

For four of seven participants with epilepsy, there was another, clinical, evidence for language laterality (Supplementary Table 4). We compared the laterality assessment of the new tasks (Figure 5; Supplementary Table 5) with that of the clinical evidence for these patients. In three patients (EP1, EP4, and EP6), the results of the new fMRI tasks were in agreement with the clinical evidence (left lateralized). For the fourth patient (EP2), the new (SR) task did not conclusively lateralize language to the left or the right hemisphere, whereas the clinical evidence indicated left-lateralized language.

## Discussion

Here we presented an fMRI approach for acquiring high-quality language laterality assessment in young children. Our data show that, using child-friendly tasks, conclusive lateralization indices can be obtained in young healthy children and in children with epilepsy.

### Comparison Between New Tasks and Classic Language Task

The new tasks developed and validated in the current study were designed specifically for children of young age, including preliterate children and children with delayed cognitive development. Not only are they easy to perform, they were also designed to attract children's attention.

In a large group of healthy participants, the SR task showed, on average, clear left-lateralized activation in the classic language areas of both the inferior frontal and the posterior temporal lobes. Notably, the strong bilateral *anterior* temporal activation can be ascribed to the role of these left and right hemisphere areas in amodal semantic processing (48). At the single-subject level, the task provided conclusive determination of hemispheric dominance in 83% of the healthy participants, 84% concordance with a well-validated fMRI task for language lateralization, and no instances of lateralization to the other hemisphere. It should be noted that story listening paradigms have been used in earlier fMRI studies to map language networks in children (49–52), but the traditional approach, using tones as a control condition, strongly activates the auditory cortex/posterior temporal cortex bilaterally, making it difficult to draw conclusions about the lateralization of posterior temporal language areas (49–53). Therefore, in the current study, we used reversed speech as a control condition. Previous studies that employed reversed speech as a contrast for forward speech reported varying degrees of activation in the posterior temporal areas and inferior frontal gyrus [e.g., (30, 31, 54–58)]. It has been suggested that active listening results in stronger inferior frontal activation than more passive paradigms (51, 53, 59). Therefore, in the current study, to make sure that children's attention was continuously drawn to the semantic content of the story and that they were actively engaged into listening to the story, we used visual support (i.e., illustrations from the children's book) and used a paradigm in which all active blocks together formed a continuous, age-appropriate, and engaging story. Our data reveal that using this approach, the SR task, a passive language task that does not require any reading skill or language production during scanning, can be used to determine hemispheric dominance in individual young healthy children. It will be interesting to investigate whether our approach is able to lateralize language also in adolescents and adults, using a story matched to the interests of that age. We propose that any SR task designed for these older groups should contain visual support as well since it may contribute to attracting attention and active listening also at higher age and since the visual input, as we used it, does not seem to produce interfering activity in the occipital visual areas.

The LPM task showed strong left-lateralized activation in the precentral gyrus and the inferior frontal gyrus, with the activation largely overlapping with that of the VG task and corresponding with earlier reports on a similar paradigm (33, 52, 60, 61). The overlap with the activation pattern of the VG task and the 63% concordance in language lateralization results in healthy participants indicate that the LPM task provides satisfactory determination of hemispheric dominance in the majority of young children. The fact that the task does not require the ability to quickly read entire words suggests that it can be used for children who are at a very early reading stage. Interestingly, the mean LPM activation pattern showed one hotspot in classic Broca's area close to the Sylvian fissure and one close to the inferior frontal sulcus. It should be noted that activity between these two hotspots was not absent but occurred somewhat deeper. Although it is currently unclear why this activity was located in deeper structures, a similar area of “inactivity” in the middle part of the inferior frontal gyrus has been shown before [(52), their Figure 1].

On average, the VG task induced a typical left-lateralized activation pattern, mostly activating not only the left inferior frontal language area but also a region in the posterior middle temporal gyrus. These findings agree with earlier reports from adults and children (33, 35, 36) and confirm that verb generation is a reliable and powerful paradigm to determine language lateralization. Our data show that it can be used successfully in healthy, literate children aged 6–10 years old, providing a conclusive determination of hemispheric dominance, yet since the task as we use it requires reading ability, the ability to produce covert speech, and the ability to quickly produce a matching verb and because it is not specifically designed to match the interests of young children, it often fails to produce good results in young patients with epilepsy. Indeed many pediatric epilepsy patients are affected in their cognitive and language function (62), and VG task performance has been shown to correlate with brain activation levels (63).

Despite the fact that all three tasks activated Broca's and Wernicke's areas to some extent, two issues are worth noting. First, the activation patterns were quite different between tasks, with LPM activating mainly Broca's area, VG showing hotspots in both Broca's and Wernicke's areas, and SR activation being more present in the temporal lobe. The different spatial activation patterns could be responsible for the differences in the LI values obtained for the tasks. For the LI computation, we used a language ROI that encompassed the inferior frontal and temporal language areas. Since all voxels in this ROI were taken into account, the LI values are expected to be lower on average for a task with less extended activity patterns (LPM) or when some of the activities are more bilaterally distributed (SR, temporal regions). Second, there was quite some variability between participants in the extent (i.e., number of voxels) of the activation. One likely cause of variability in the activation between participants is the level of head motion. In addition, inter-subject differences in (the development of) brain anatomy may contribute to this variability (64). Unfortunately, our current healthy sample does not allow to draw conclusions on this topic or to evaluate if the variability exceeds the norm.

Taken together, our data show that each of our new tasks is able to determine language lateralization in young healthy children aged 6–10 years, with the SR task providing conclusive lateralization more frequently than the LPM task. Given that they can be performed also by children who cannot yet read words (adequately), they may be especially suitable for use in children of even younger age or children with delayed cognitive development and may become of relevance for the area of pediatric epilepsy surgery, where an increasing number of young children is treated (3). Indeed the SR task and the LPM task were completed by six of seven and five of five participants with epilepsy, respectively (two other participants with epilepsy did not perform the LPM task due to time constraints). In contrast, only three of seven completed the VG task (two of whom the slow version). The four others were unable to perform the VG task at all or did not complete the run despite the fact that the VG task was the shortest of the three tasks. This indicates that we did succeed in designing tasks that are engaging for young children, a critical feature for obtaining functional topographical information for treatment planning. Conclusive LI values were obtained with the new tasks in most participants with epilepsy, in particular, also in the two patients who were unable to perform the VG task. The high percentage of conclusive language lateralization obtained with the VG task (92% in healthy participants and 80% in participants with epilepsy), however, indicates that, whenever children are able to read and produce words at a decent pace, the fMRI task set should include this task, ideally backed-up with one or both of the new tasks studied here. In cases where children are unable to perform the VG task, however, our data suggest that fMRI scanning to assess language laterality may be feasible with the new tasks.

### Participant Preparation

Thorough preparation of (pediatric) study participants or patients before (f)MRI scans has been addressed previously. Besides preparatory videos or images (65), the use of a mock-scanner has been indicated to reduce the need for sedation and the levels of distress among children and adolescents and has been suggested to positively contribute to the acquisition of high-quality (f)MRI images of young children (66–71). Here we show that a mock-scan session significantly reduces the levels of anxiety and increases enjoyment in young healthy children with limited or no previous fMRI experience, as rated by the participants themselves, their parents, and a researcher. These data suggest that, besides the previously reported positive effects of a mock-scan session on data quality, mock-scanner preparation also improves participant or patient experience. The levels of anxiety and enjoyment the healthy participants reported after the real fMRI scan did not differ significantly from those immediately after the mock-scanner practice, and for anxiety, remained significantly different from the pre-mock-scan values. This suggests that the mock-scanner practice effects on anxiety extend to the real fMRI scan. In the current study, most patient participants had extensive experience with MRI scans and therefore did not conduct a mock-scan session. We therefore do not know whether or not also in the more experienced population a mock-scan session could have a beneficial effect, and this is an interesting topic for further investigation. Yet given the benefits of a mock-scan session for the cost-effectiveness of (f)MRI scans, the duration of clinical and diagnostic trajectories (both by minimizing the need for anesthesia or repeated scans), and participant or patient experience, we believe that a mock-scan session should be part of the standard research and clinical routines for preparing young children before an (f)MRI scan, especially in those who are naive to the MRI setting.

### Limitations

Several limitations apply to the current study. First, the current tasks were part of a larger study in which other brain functions were also addressed. Therefore, the list of fMRI tasks was too long to be performed by a single participant. For these reasons, not all language tasks were performed by all participants. Although this did not lead to a bias with respect to age or gender per task, not all participants could be included in the within-subject comparison of the lateralization indices. Second, our ability to acquire adequate data for large percentages of healthy children may be biased by the fact that parents of more fidgety children or those with a relatively short attention span may have been more reluctant to propose their children for participation. Third, the active and the rest conditions of the language tasks used were not perfectly balanced in terms of visual input. Indeed for the VG and the LPM tasks, we observed activation in the occipital areas, indicating increased visual processing in the active condition, yet given the large distance between the language areas of interest and the occipital activation, we believe that this phenomenon does not affect our conclusions. Fourth, the tasks used were different in their design and duration, and it may therefore be expected that they differ in their sensitivity to activate the language network. Yet because of the conceptual differences of the tasks (e.g., language perception vs. language production), differences in activation may be expected even in the case of exactly same design and duration. Since our aim was not to quantitatively assess which of the tasks provides the strongest activation in the language areas but rather to determine if our new tasks allow for the generation of high-quality language maps in young children, we believe that differences in task duration and design are, although statistically suboptimal, secondary to the goal of maintaining attention throughout the task. Notably, the variable task duration did not lead to significant differences in the percentage of scans that had to be removed due to excessive head motion, which indicates that there is a relatively consistent and systematic loss of number of scans per unit of time. Finally, the size of our clinical sample of participants with epilepsy was small and did not include children with confirmed atypical language lateralization. Further validation of the new approach in the target population will be important and should focus on determining the sensitivity of the new tasks to detect bilateral or right-lateralized language representation.

## Conclusion

In conclusion, our data show that, with tasks designed to match the interests and the abilities of young children, reliable fMRI language mapping can be obtained in healthy young children and in children with epilepsy. Since the tasks developed here can be performed by children who are unable to read words (adequately), the approach described in this manuscript may contribute to the determination of hemispheric dominance in the increasing number of young children scheduled for epilepsy surgery.

## Data Availability Statement

The datasets presented in this article are not readily available because sharing of the data requires consent of the participants. Requests to access the datasets should be directed to Mariska J. Vansteensel, m.j.vansteensel@umcutrecht.nl.

## Ethics Statement

The studies involving human participants were reviewed and approved by METC Utrecht. Written informed consent to participate in this study was provided by the participants' legal guardian/next of kin.

## Author Contributions

LC, KB, NR, and MJV designed the study. LC, PC, and MJV acquired the data. LC, MR, MV, and MJV analyzed the data. LC and MJV drafted the manuscript. All authors interpreted the data and revised the manuscript.

## Conflict of Interest

NR was a shareholder in Braincarta, a clinical fMRI company. Braincarta was not involved in this study. The remaining authors declare that the research was conducted in the absence of any commercial or financial relationships that could be construed as a potential conflict of interest.
