# Supplementary material for: A Functional Magnetic Resonance Imaging Approach for Language Laterality Assessment in Young Children
Source: Front Pediatr. 2020 Nov 17;8:587593. doi: 10.3389/fped.2020.587593 (PMC7707083; doi:10.3389/fped.2020.587593)
Supplement: Supplementary file 1 [file Data_Sheet_1.pdf]

Supplementary Material

Nr	Age	Gender	Handed-ness	Anatomy	SR	LPM	VG
HP1	6.10	f	R	1	1	n/a	n/a
HP2	6.15	f	R	1	n/a	1	n/a
HP3	6.35	f	L	1	1	1	1**
HP4	6.70	f	R	1	1	n/a	n/a
HP5	6.78	f	R	1	1	1	1
HP6	6.83	m	R	1	1	1	1
HP7	6.89	f	R	1	1	1	n/a
HP8	7.34	f	R	1	1	n/a	1
HP9	7.63	f	L	1	n/a	1	1
HP10	7.64	m	L	1	n/a	1	n/a
HP11	7.70	f	R	1	1	1	1
HP12	7.93	m	R	1	1	n/a	1
HP13	8.18	m	R	1	n/a	1	1**
HP14	8.24	f	R	1	1	n/a	1
HP15	8.52	m	R	1	1	1	1
HP16	8.61	f	R	1	1	1	n/a
HP17	8.71	m	R	1	1	1	1
HP18	8.96	m	L	1	1	1	1
HP19	9.02	f	R	1	1*	1	1
HP20	9.07	f	R	1	n/a	1	1
HP21	9.19	f	R	1	1	1	1
HP22	9.35	m	R	1	1	1	1**
HP23	9.39	f	R	1	1	1	1
HP24	9.62	m	R	1	1	1	1
HP25	9.65	m	R	1	1	1	1
HP26	9.72	m	L	1	1	n/a	1
HP27	10.21	f	R	1	n/a	1	1
HP28	10.40	m	R	1	1	n/a	1
HP29	10.70	m	R	1	1	1	1
HP30	10.95	m	R	1	n/a	1	1
		**16f/14m**	**25R/5L**		**20R/3L**	**19R/4L**	**20R/4L**

*Supplementary Table 1. Demographics and acquired scans per healthy participant. Left handed participants are indicated by grey shading. 1=task/scan acquired, n/a = task/scan not acquired, *2/3 of the task was acquired, **slow version of VG task, see methods section for details.*

		Percentage scans removed			Proportion variance remaining scans		
**Number**	**Age**	**SR**	**LPM**	**VG**	**SR**	**LPM**	**VG**
1	6.10	12.6			0.88	-	-
2	6.15	-	68.4		-	0.30**	-
3	6.35	45.5	9.9	61.2	0.54	0.90	0.38**
4	6.70	5.8	-	-	0.94	-	-
5	6.78	21.6	8.2	5.6	0.76	0.92	0.95
6	6.83	4.0	6.5	1.9	0.97	0.93	0.98
7	6.89	10.1	14.0	-	0.89	0.86	-
8	7.34	0.0*	-	0.2	1*	-	1.00
9	7.63	-	14.3	2.1	-	0.85	0.98
10	7.64	-	27.5	-	-	0.73	-
11	7.70	11.0	4.0	4.4	0.90	0.96	0.94
12	7.93	7.6	-	11.1	0.94	-	0.90
13	8.18	-	62.0	44.6	-	0.39**	0.56
14	8.24	9.5	-	7.0	0.89	-	0.93
15	8.52	18.2	6.6	11.4	0.80	0.94	0.86
16	8.61	45.0	18.4	-	0.50	0.81	-
17	8.71	1.3	50.1	1.3	0.99	0.49	0.99
18	8.96	2.7	0.6	1.7	0.97	0.99	0.98
19	9.02	7.4	6.8	5.0	0.91	0.93	0.95
20	9.07	-	2.2	2.1	-	0.98	0.98
21	9.19	1.1	0.4	0.2	0.99	1.00	1.00
22	9.35	3.3	8.2	12.1	0.97	0.92	0.85
23	9.39	0.8	0.0*	0.0*	0.99	1*	1*
24	9.62	53.9	2.5	7.7	0.46	0.98	0.93
25	9.65	19.7	17.6	27.6	0.79	0.82	0.68
26	9.72	13.7	-	10.3	0.87	-	0.91
27	10.21	-	3.8	0.2	-	0.97	1.00
28	10.40	0.9	-	2.5	099	-	0.98
29	10.70	23.3	16.7	3.3	0.75	0.83	0.97
30	10.95	-	22.6	23.7	-	0.76	0.76

*Supplementary Table 2. Percentage of removed scans (by motion filter, due to above-threshold motion) and proportion of statistical power (PSP) remaining after removal of the contribution of volumes with above-threshold motion. *0% scans excluded from analyses, **PSP lower than 0.4, dataset removed from further analysis.*

Lateralization Index				
**Number**	**Age**	**SR**	**LPM**	**VG**
1	6.10	0.51	-	-
2	6.15	-	Excl	-
3	6.35	0.12	0.23	Excl
4	6.70	0.90	-	-
5	6.78	0.69	0.19	0.64
6	6.83	0.65	0.76	0.82
7	6.89	0.13	0.64	-
8	7.34	0.82	-	0.73
9	7.63	-	-0.51	-0.82
10	7.64	-	0.47	-
11	7.70	0.33	0.14	0.76
12	7.93	0.79	-	0.65
13	8.18	-	Excl	0.16
14	8.24	0.37	-	0.41
15	8.52	0.44	0.66	0.73
16	8.61	0.31	0.02	-
17	8.71	0.75	0.37	0.92
18	8.96	0.36	0.63	0.73
19	9.02	0.31	0.29	0.86
20	9.07		0.14	0.44
21	9.19	0.39	0.75	0.83
22	9.35	0.52	0.34	0.46
23	9.39	0.77	0.56	0.77
24	9.62	0.85	0.43	0.42
25	9.65	0.50	0.67	0.60
26	9.72	0.54	-	0.65
27	10.21	-	0.39	0.94
28	10.40	0.09	-	0.79
29	10.70	0.14	-0.14	0.55
30	10.95	-	0.16	0.55
		n=23<br>0 excl	n=23<br>2 excl	n=24<br>1 excl

Supplementary Table 3. Lateralization indices of all healthy participants and all language tasks. Excl = dataset excluded from analysis because of excessive movement (PSP value <0.4). At the bottom, the total number of datasets per task is given, and the number thereof that was excluded due to excessive motion.

Nr	Age	Gender	Handed-ness	Seizure focus	MRI results	Clinical Language Laterali-zation	Anatomy	SR	LPM	VG
EP1	7.51	f	R	R temporo-occipital	Suspected multilobar type 1 dysplasia R	L (fTCD)	1	1	n/a	1***
EP2	7.99	m	R	R temporal	Negative (pathology: hippocampal sclerosis)	L (fTCD)	1	1	n/a	1**
EP3	8.12	m	R	L parietal	L parietal focal	n/a	1	1	1	n/a*
EP4	8.63	m	L	L central	L central ischemic injury	L (ECS)	1	1	1	n/a*
EP5	8.88	m	R	R precentral	R precentral focal cortical dysplasia	n/a	1	1	1	1**
EP6	10.65	m	L	Bilateral temporal	Normal	L (wada)	1	1	1	1
EP7	11.10	f	R	R frontotemporal	Large R middle cerebral artery infarct	n/a	1	1***	1	1***

*Supplementary Table 4. Demographics and acquired scans per epilepsy participant. Left handed participants are indicated by grey shading. 1 = task/scan acquired, n/a = task/scan not acquired, fTCD = functional transcranial doppler, ECS = electrocortical stimulation, *the participant was unable to perform the normal or fast version of the VG task, **slow version of VG task, see methods section for details, ***task suspended prematurely on participant request (task at least 85% complete).*

**Lateralization Index**				
**Number**	**Age**	**SR**	**LPM**	**VG**
EP1	7.51	0,81	-	0,25
EP2	7.99	0,14	-	0,31
EP3	8.12	0,62	0,31	-
EP4	8.63	0,40	-0,13	-
EP5	8.88	-0,15	0,35	0,19
EP6	10.65	0,21	0,46	0,23
EP7	11.10	0,39	0,41	0,73
		n=7<br>0 excl	n=5<br>0 excl	n=5<br>0 excl

Supplementary Table 5. Lateralization indices of all epilepsy participants and all language tasks. At the bottom, the total number of datasets per task is given, and the number thereof that was excluded (excl) due to excessive motion.
